# Magnetic Resonance Simulation in Education: Quantitative Evaluation of an Actual Classroom Experience †

**DOI:** 10.3390/s21186011

**Published:** 2021-09-08

**Authors:** Daniel Treceño-Fernández, Juan Calabia-del-Campo, Fátima Matute-Teresa, Miguel L. Bote-Lorenzo, Eduardo Gómez-Sánchez, Rodrigo de Luis-García, Carlos Alberola-López

**Affiliations:** 1 ETSI Telecomunicación, University of Valladolid, 47011 Valladolid, Spain; dtrefer@lpi.tel.uva.es (D.T.-F.); migbot@tel.uva.es (M.L.B.-L.); edugom@tel.uva.es (E.G.-S.); rodlui@tel.uva.es (R.d.L.-G.); 2 Hospital Clínico Universitario, 47003 Valladolid, Spain; juancalabia@gmail.com; 3 Hospital Clínico San Carlos, 28040 Madrid, Spain; fatimamatuterx@gmail.com

**Keywords:** magnetic resonance imaging, MRI, simulator system, medical training/educational tool, radiographer training

## Abstract

Magnetic resonance is an imaging modality that implies a high complexity for radiographers. Despite some simulators having been developed for training purposes, we are not aware of any attempt to quantitatively measure their educational performance. The present study gives an answer to the question: Does an MRI simulator built on specific functional and non-functional requirements help radiographers learn MRI theoretical and practical concepts better than traditional educational method based on lectures? Our study was carried out in a single day by a total of 60 students of a main hospital in Madrid, Spain. The experiment followed a randomized pre-test post-test design with a control group that used a traditional educational method, and an experimental group that used our simulator. Knowledge level was assessed by means of an instrument with evidence of validity in its format and content, while its reliability was analyzed after the experiment. Statistical differences between both groups were measured. Significant statistical differences were found in favor of the participants who used the simulator for both the post-test score and the gain (difference between post-test and pre-test scores). The effect size turned out to be significant as well. In this work we evaluated a magnetic resonance simulation paradigm as a tool to help in the training of radiographers. The study shows that a simulator built on specific design requirements is a valuable complement to traditional education procedures, backed up with significant quantitative results.

## 1. Background

Magnetic Resonance Imaging (MRI) is a non-invasive medical imaging modality commonly used for diagnosis of pathologies related to soft tissue and has experienced great growth in recent years, as reflected in [1]. MRI offers excellent contrast in soft tissue with non-ionizing radiation and is extremely versatile since a myriad of image contrasts can be obtained by setting its many available parameters.

Unfortunately, this flexibility bears a cost. Radiologic technologists (i.e., radiographers) need a deep background in this technique to sort out all the difficulties they come across in daily practice. In addition, their duty is carried out in a highly demanding clinical environment, where both image quality and patient throughput are to be maximized; this has the consequence of minimum (or null) scanner time allocation for training. Moreover, the software installed in these machines poses additional difficulties, even for experienced radiologists [2].

MRI computer simulators are a natural alternative for radiographer training; a variety of these systems has been proposed both for educational end research purposes. About the former, we can mention four contributions. The Bloch Simulator, described in [3], is a simulator designed to explain basic concepts about the magnetic resonance principle such as the reference frames or the spins’ dephasing and rephasing. The tool has a number of parameters that can be changed and the user can observe the consequences of these changes, but no clinical workflow is observed. The technology used is Adobe Flash Player. Simplified MRI was described in [4] and it is intended as a learning help for quantum mechanics. In terms of usage, similar considerations as those discussed for the Bloch Simulator apply; in terms of technology, it is provided as a Java virtual machine. Virtual MRI, proposed in [5], is a simulator that mimics some functions and features available in an actual MR scanner. Several pulse sequences are available; the main involved parameters can be changed and motion artifacts can be introduced; images can be observed on both k-space and image space. However, no 3D geometrical planning is available and simulations for some sequences seem to take quite long for an educational tool; this may be due to the fact that it is a Java-based application. Finally, the Torheim Simulator [6] has some similarities with Virtual MRI and it allows the user to add some sort of simulated pathology. It was developed in C++ about ten years before Virtual MRI.

Simulators intended for research purposes have also been described. Here is a brief description of those considered more relevant. MRILab was proposed in [7] and has been since continuously evolving [8]. It is quite a complete MR simulator, with several panels that provide a wide spread of funcionality, such us gradient types, different coils and sequences etc. However, although FOV selection is available, geometrical planning is limited to orthogonal planes. The application runs in Matlab in terms of user interface although the simulation kernel runs in C/C++ and some CUDA functionality is also available. Another popular simulator is JEMRIS [9]; JEMRIS is a general-purpose simulator that enables the user to simulate off-resonance effects, gradient non-linearities, different coil geometries and additional effects such as chemical shift. MPI technology is used for parallel computing albeit GPU devices are not directly usable since the simulation kernel is written in C++, targeting CPU exclusively. Matlab is also used for graphical interfacing, although no advanced geometrical planning seems available. More recently, functionality has been included to feed real scanners with JEMRIS sequences [10] as well as to simulate flow [11]. SIMRI was proposed in [12] and it also provides the solution to the Bloch equations for the simulation of a number of sequences. Parallel programming is also available as well as some graphical interfacing facilities for 1D signal visualization and 2D spin evolution. Emphasis in fast resolution of the Bloch equations is also made in MRISIMUL [13] where CUDA functions have been developed for cardiovascular acquisitions. Similar considerations can be done with respect to PSUDOMRI [14] and BlochSolver [15]. SpinBench is another Bloch equations solver which includes additional graphical representations of the magnetization evolution. ODIN [16] is a windows-oriented simulator that has been used, among others, form SPECT/MRI simulation and for pulse design. Several windows provide different functionality, such as parameter setup, sequence configuration, slice selection and visualization of simulation dynamics. Some sort of geometrical planning exists, but this simulator does not mimic the procedure used in clinical practice, but the interaction is limited to slider movement. Finally, some specifically-targeted simulators have been proposed, such as POSSUM [17], which is part of the well-known FSL library (https://fsl.fmrib.ox.ac.uk/fsl/fslwiki, last access on 30 July 2021) and it was originally focused in fMRI (although later has given room to other domains, such as diffusion [18,19]), or simulation of magnetic resonance angiography [20].

Recently, our team has developed an MRI simulator [21] that allows practitioners to mimic the workflow used for routine MR acquisition. As indicated above, educational simulators have focused in illustrating specific features about the magnetic resonance phenomenon but not so much in replicating the steps customarily taken by a radiographer in daily practice. A very relevant action, such as spatial planning, is usually overlooked. In addition, we intended to provide a solution that could run on-line from a learning management system, with no installation needs, and with platform independence. This would allow us to offer both classroom-oriented and on-line courses, as well as to update our simulator versions transparently to the user. Hence, our solution is based on Web technologies. For interactivity purposes as an educational tool, simulations should be characterized by speed even although image quality could be slighty compromised. This simulator has been complemented with an intelligent tutoring system for additional hands-on guidance [22].

Despite MRI simulators seem a valuable resource for radiographers training, no attempt has been made, to the best of our knowledge, to evaluate their impact in an educational process; hence, the question about whether an MRI simulator is a valuable educational tool remains formally unanswered. In this paper we provide guidelines to design an MRI simulator intended for educational purposes and we quantitatively answer the question referred to above by means of an actual classroom experience.

## 2. Methods

The question we intend to answer is: Does an MRI simulator that follows our design requirements—as defined in Section 2.1—help radiographers learn MRI theoretical and practical concepts better than traditional teaching methods (i.e., lectures supported by slides)?

This section describes the materials and methods employed to reach this goal.

### 2.1. The MRI Simulator

In this section we provide a brief overview of the MRI simulator described in [21]; additional information can be found in that reference. Specifically, we have identified a set of features that an MRI simulator designed for radiographer training should possess; the requirements stem from both literature review and interviews with senior radiologists from the Spanish Society of Medical Radiology (SERAM, https://www.seram.es/, last access on 30 July 2021), and radiographers and educators from the School of Radiographers of the Hospital Clínico San Carlos, Madrid, Spain. Functional requirements are listed as follows:

- The system should be able to simulate images created from a set of acquisition sequences that constitute a protocol. The user should also be able to create and execute those protocols. Patient positioning and coil selection should also be available.
- The user should be able to change basic acquisition parameters, such as $T_{E}$ (echo time), $T_{R}$ (repetition time) and, where applicable, $T_{I}$ (inversion time), flip angle, ETL (echo train length) and others.
- Geometrical planning should be included in the simulation workflow, from slice orientation to the determination of the FOV (field of view), slice thickness, slice separation, and selection of phase/frequency encoding directions.
- Acquisition artifacts should be generated at trainer demand.
- k-space manipulation should be supported.
- Different educational roles should be supported, allowing trainers to create educational scenarios and trainees to work on those scenarios and report their results.

Non-functional requirements are:

- Short simulation times are needed so that action/reaction is possible in acceptable times for an educational session.
- The system should be easy to access/install and able to work over a wide range of platforms.
- The system will avoid, whenever possible, the specificities associated to each manufacturer as well as to use vendor-associated sequence names.

#### 2.1.1. Architectural Design and Technologies for Implementation

Based on foregoing requirements, we have opted for a simple simulation model, consisting in evaluating mathematical expressions of well-known sequences which are then corrupted with artifacts; the simulation model is described in more detail in Section 2.1.3. Rigorous detailed simulations, some of which are referred to in Section 1, have been avoided to allow a more responsive user experience. As for ease of access and installation, we have opted for a Web-based application.

**Figure 1:**
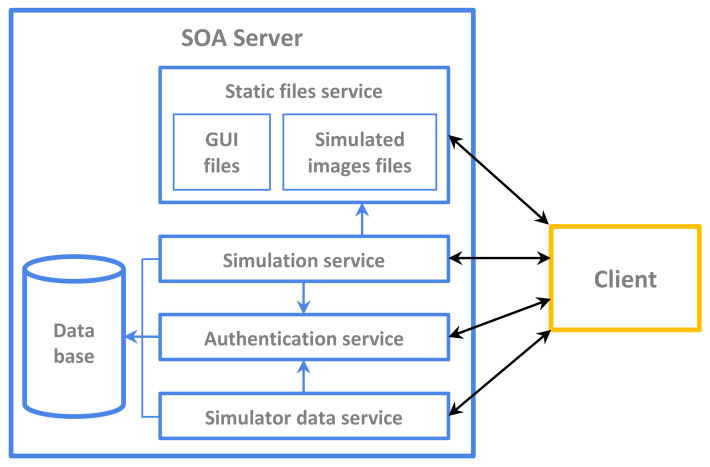
Service-oriented architecture (SOA) Server.

The system has been designed following a client-server architecture; the server, follows a service-oriented architecture (SOA), where services are depicted in Figure 1. The simulation service (Figure 1) uses the simple object access protocol (SOAP) given the need to exchange rich requests and responses with the client. The remaining services apply a representational state transfer (REST) application program interface (API).The server has been programmed in Python using the Django framework [23]. Simulations are performed using C++ and the ITK library; interaction with Python is achieved through a wrapper.

On the client side, the interface has a component-oriented design, where each interface element consists of one or several components that follow the Model–View–Viewmodel (MVVM) pattern [24]. The graphical user interface (GUI) has been implemented with AngularJS.

#### 2.1.2. Interface Overview

**Figure 2:**
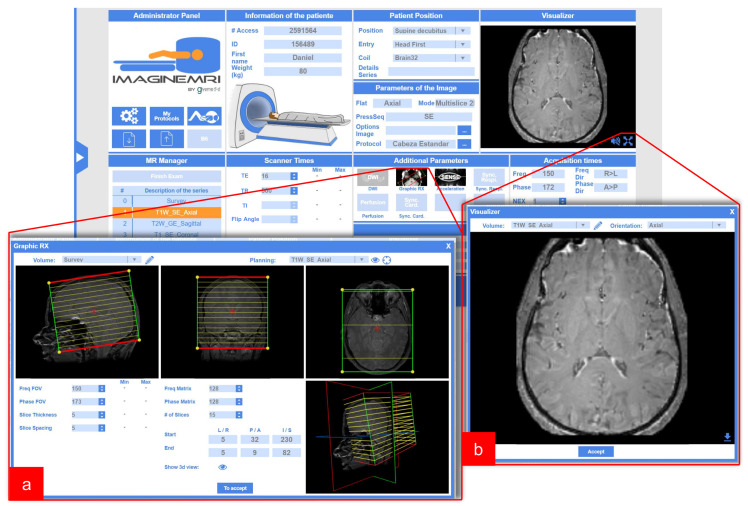
Web-based MRI Simulator. Image of the main panel of the simulator and two pop-up panels: (**a**) The panel for the location of the slices and (**b**) the viewer of the images obtained.

The simulator interface has been designed to mimic the interface of an actual MRI console, as well as to provide support to all the necessary steps that the user needs to perform to run the simulation; we have meant to be generalist in order to comply with our third non-functional requirement. A general view of the main interface is provided in Figure 2. On this interface, the user needs to: Enter patient information, select patient position and coil and select a protocol from a menu. Then, a protocol should be loaded and the interface will display each of the pulse sequences comprised. As a general rule, for each sequence the user should, on the one hand, select the relevant parameters through panels and, on the other, carry out the appropriate geometrical planning (Figure 2a), which is done graphically. Finally, the scan button should be pressed; at this moment the data of the interface is sent to the server, which will carry out the simulation and return a volume for its visualization (Figure 2b). In addition, more advanced options can be used, such as no phase wrap, shimming and saturation bands.

Some other panels offer additional functionality; this is the case of a menu that permits the visualization of different pathological cases as well as a panel to activate/deactivate different image artifacts, to select different hardware properties (field strength or field inhomogeneity), and to choose a specific case to be simulated (different anatomical regions and/or different pathologies).

#### 2.1.3. Simulation Overview

**Figure 3:**
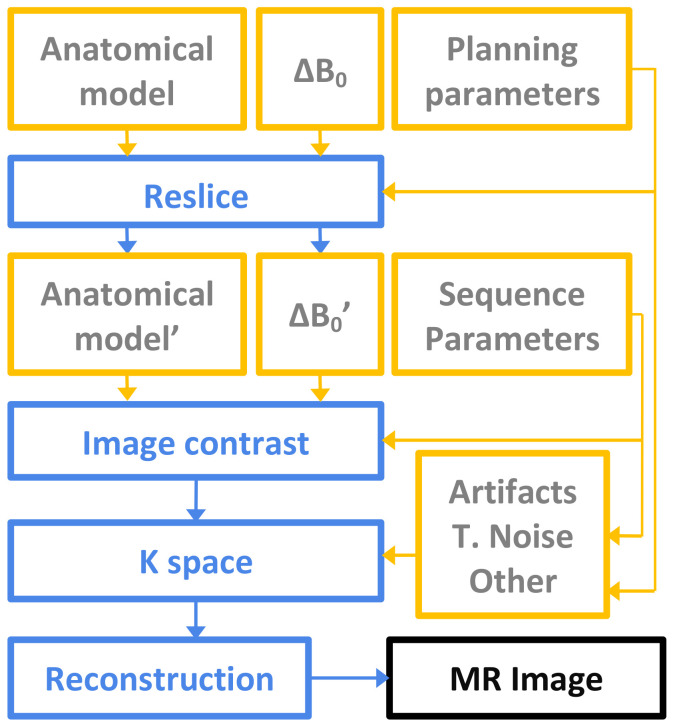
Block simulation scheme.

A diagram depicting the pipeline of the processing steps required for the computation of the simulated MRI images is shown in Figure 3. The procedure is as follows:

The initial ingredients that are necessary to perform the simulation are the anatomical model, the magnetic field inhomogeinity ($\Delta B_{0}$), the geometrical planning parameters and the sequence parameters. The anatomical model consists of a set of 3D volumes that contain the tissue properties needed for MR simulation, namely, proton density (PD), longitudinal relaxation time ($T_{1}$) and transverse relaxation time ($T_{2}$). $\Delta B_{0}$ is a synthetic perturbation since we only mean to highlight the need of a shimming procedure prior to the actual acquisition (i.e., of a self calibration for homogenizing the field).

Geometrical planning parameters are specified by the user and they determine both the resolution and the FOV (field of view) of the image. Sequence parameters fully define a specific sequence, and include the $T_{E}$, $T_{R}$, flip angle and $T_{I}$ when appropriate and/or ETL. See Appendix A for further details.

Starting from the anatomical model and $\Delta B_{0}$, a reslice operation is first performed, which creates volumes of the size and properties specified by the user by means of the chosen geometrical planning parameters. Next, image contrast is simulated from these volumes by evaluating well-known algebraic expressions corresponding to the specific sequence chosen by the user [25,26]. Afterwards, a Fourier transform is applied to the contrast image, yielding the so-called k-space. In this space a number of artifacts can be easily simulated, such as motion effects and spike or thermal noise. In addition, attenuation effects from fast sequences are also incorporated, together with any other k-space manipulation options (half Fourier reconstruction, for instance). Finally, the inverse Fourier transform is computed to generate the resulting simulated MR image. This image will also contain additional spatial information (orientation, origin and voxel size) for its correct visualization.

### 2.2. Participants

Participants were recruited among students enrolled in two degrees from the School of Radiographers at the Hospital Clínico San Carlos, Madrid, Spain. From a cohort of 80 students, 64 of them volunteered to participate in the experiment. Most students had no previous specific knowledge on MRI except for a group of 30 students, who had received a 4-h shallow introduction to the topic.

### 2.3. Experimental Design

The experimental setup was designed in collaboration with faculty members of the above-mentioned school; the educational experience was carried out in one single day during the regular schedule. With this choice we intended both to maximize the number of attendees as well as to avoid interaction (and, hence, contamination) of the two groups in which we split the students (see below).

**Figure 4:**
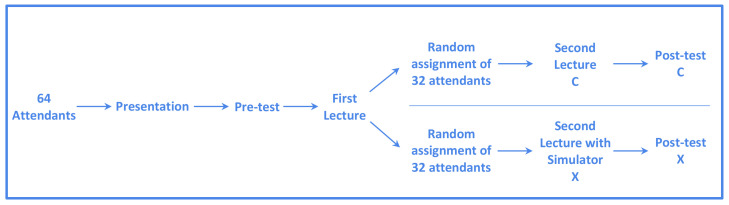
Study Design. Letter X refers to the experimental group, while C refers to the control group.

As depicted in Figure 4, the experiment followed a randomized pre-test post-test design with a control group (CG) and an experimental group (EG) [27]. The steps are now described in sequence:

- An introductory lecture was given to all participants, where the essentials of the experimental design were explained. We clearly stated its optional character and students were guaranteed of anonymity preservation, and null effects on this experiment in their final grades. Then, participant written consents were collected, following the university ethics standards.
- The pre-test was performed using the instrument that is described in the Section 2.4.
- A 90-min lecture supported by slides was given to all the participants; the covered topics were: Magnetic properties of the tissues, concept of magnetic resonance, pulses and gradients in MRI, the k-space formalism and image formation, spin-echo (SE) and gradient-echo (GE) sequences and safety guidelines.
- Students were randomly assigned to the EG/CG. The EG was guided to a computer room located in a nearby building by the former, while the CG remained in the classroom and was awarded a short break in order to allow a perfect synchronization between both groups. Then, a 90-min lecture was given to both groups including the following topics: k-space formation, relevant time parameters in MRI sequences (mainly, $T_{E}$ and $T_{R}$), geometrical planning and related parameters, and image artifacts. In the CG, these topics were covered by means of a slides presentation where the effects of parameter choices were illustrated. In the EG, participants employed the MRI simulator through hands-on guided exercises to see the topics.
  Both lectures contents were agreed with the school faculty as a trade-off between the topics that could be covered by the simulator and the expected learning outcomes of the school in terms of magnetic resonance imaging. The material used for preparing these contents were both well-known academic references [25,26] and popular web sites related to MRI fundamentals (https://mrimaster.com/, http://mriquestions.com/, http://xrayphysics.com/ the three of them last accessed on 30 July 2021).
- The post-test was given to both groups.

Two trainers were involved in the lectures; one of them was in charge of the first lecture to all the students and the second lecture to the EG, while the other was responsible for giving the second lecture to the CG. In order to avoid any bias between the two groups, the detailed content of the second lecture was agreed on beforehand between the two trainers and covered each of the questions asked in the practical part of the post-test; in addition, the two trainers were in communication to synchronize session start and ending.

There were a few minor incidents during the experiment: (a) Two students from the CG and another student from the EG did not attend the second lecture; (b) one student in the EG deliberately answered “I do not know” to all questions of the post-test; this participant was excluded from the analysis. Overall, the number of students who completed the experiment was 30 for each group.

### 2.4. Measure Instrument

To determine the level of knowledge in MRI, a 20-item questionnaire was designed. Following [27], experts were involved in the creation of the measure instrument to assure its validity. Specifically, both the aim and the content of the questionnaire were explained to two different faculty members of the radiographers school. They were asked to analyze whether the questionnaire items could be considered an adequate sample of the lecture contents; the items were iteratively refined until all of them were approved.

Appendix B shows the questionnaire translated into English. Each item has four possible answers together with the answer “I do not know”; the latter intends to avoid random answers. The first 10 items correspond to the content of the first lecture, as enumerated in Section 2.3, while the remaining items correspond to the content of the second lecture. Since the content of this first part deals with theoretical aspects of MRI and was presented in a purely explanatory way, we will hereafter refer to it as “theoretical” part or “T part”, while the second will be hereafter referred to as “practical part” or “P part”. The score of the questionnaire is the number of correct answers (hereafter referred to as “hits rating”); hence the maximum score is 10 points in each part. This score will be used by default. For the sake of completeness, a null-expectation version of the rating—random answering leads to an average zero score—has also been accounted for.

Given the nature of our experiment, it was not possible to employ a measure instrument whose reliability was tested beforehand. Therefore, the reliability of the instrument was computed post hoc, as described below.

### 2.5. Statistical Analysis

The instrument reliability was computed by means of the Kuder-Richardson Formula KR-20 [28] for the hits rating while we used the Cronbach’s alpha [29] for the null-expectation rating.

The statistical inference was performed as follows: A Shapiro–Wilk [30] normality test was used on the pre-/post- test scores of each group to determine whether a t-test or Wilcoxon signed rank test should be employed. Bilateral unpaired tests were run, where the null hypothesis is “Scores coincide for both groups”, while the alternative hypothesis is “Scores do not coincide”. Median, mean, standard deviation and effect size (calculated using Cohen’s d [31]) were used for the descriptive analysis. Computations were carried out in R.

The analysis followed this path: First, the test reliability was checked. Then, we tested whether both groups indeed departed from the same rates in the pre-test so that any differences in the post-test could be considered a result of our intervention. Differences between both groups in the post-test both in terms of the score as well as in terms of the gain—defined as the difference between the scores of the post-test and the pre-tests—were then tested.

## 3. Results

As for reliability, the KR-20 values in the post-test were 0.446 in the T part and 0.318 in the P part, which are too low so as to draw any further conclusion. Consequently, we carried out a correlation analysis aimed at identifying a set of questions which would provide us with higher reliability [32]. As a result of this analysis we gave rise to a 10-item instrument, selecting items {4,5,6,8,10} from the T part (recall Appendix B) and items {11,12,13,17,20} from the P part; the maximum score is now 5 points in each. The reliability obtained for this new instrument in the pre-test was 0.592 in the T part and 0.432 in the P part, while in the post-test it was 0.604 in the T part and 0.570 in the P part.

Table 1 shows the statistical analysis of the pre-test for both ratings, the correct answers and the null expectation; despite sampling differences between both groups are appreciated in the mean values, differences are not significative so we can conclude that both groups depart from a balanced situation. The first half in Table 2 shows the results corresponding to the post-test for the hits rating (labelled as `Hits’). Specifically, its first numerical column shows the *p*-values of the hypothesis test about the equality of the scores of both groups; significative values have been boldfaced. Therefore, differences in the P part of the instrument between the EG and the CG are appraised, whereas the two samples do not show differences as for the T part. Notice that the EG passes the practical part with this score procedure—scores should be multiplied times 2 to reach a scale on in the range (0,10) points and more than half of the maximum points are achieved—. The second half in Table 2 shows that the same trends are observed for null expectation (NE) rating. Interestingly, for this alternative rating the same questions as in the former rating turned out to be selected to maximize reliability. In addition, the Cronbach’s alpha exceeded 0.5 in both the T and the P parts of the post-test and no differences were again appreciated in the pre-test between the groups.

## 4. Discussion

A KR-20 reliability value of or above 0.7 is usually taken as good for instruments in the research field, although in more demanding tests [33] established acceptable reliability is above 0.5. Our initial 20-item instrument did not reach this level in the post-test; however, the 10-item subset did achieve this requirement for the post-test, as shown in Section 3. We should stress that reliability is penalized by randomness in the answers, which may explain the low values in the P part of the pre-test; notice the low scores obtained by the students in the pre-test and, specifically, in its P part (Table 1).

Table 1 shows that our study departs from a balanced situation with the 10-item instrument, i.e., the two groups have no statistical differences in their levels of expertise. Table 2 summarizes the results of our intervention. Specifically, central tendency measures reflect how the score of the EG in the post-test and in the gain is higher in the P part than the CG; as for the T subset, slightly better results are observed for the CG. However, statistical inference shows that the results of the P part are indeed significant in favor of the EG, both for the test scores and for the gain, wheareas no significant differences are observed for the T part in any of these two dimensions. This is accompanied with significant values—above 0.5—of the effect size, as measured by Cohen’s d, for the P test and the gain. All these results let us state that the MRI simulator is indeed a valuable tool for training MRI technologists when it comes to understanding practical concepts.

As for the theoretical dimension, despite results are inconclusive, we may interpret that the CG may have received a higher emphasis on the theoretical part since the second lecture did not include direct student hands-on work, which is a time consuming process; this amount of class-room time, which was employed by the students in the EG to gain familiarity with the tool, was spontaneously used by the instructor of the CG to emphasize background concepts.

We may also highlight that hypothesis tests have been used in their bilateral form; their unilateral counterparts do provide more pronounced differences. Interestingly, neither the T score nor the T gain are significant when the test is run as unilateral with the opposite alternative hypothesis (*p*-values in this case are 0.27 and 0.145 respectively). Consequently, we have reported quite conservative results, which make us confident of our findings. Once again, if the NE rating is used instead, similar trends are perceived in all the measured dimensions, as indicated in Table 2.

In this paper we have only reported the results that have allowed us to compare CG and EG. For the sake of completeness, it is worth mentioning that we have also analyzed our tool in terms of student satisfaction in three actual educational experiences; one of them was the one described in this paper and the other two were on-line, by means of two 50-h on-line courses endorsed by the SERAM, which took place in the interval May–July of 2018 and 2019, respectively. In the three experiences we used the System Usability Scale (https://www.usability.gov/how-to-and-tools/methods/system-usability-scale.html, last access on 30 July 2021), which we complemented with an enquiry about the simulator Likelihood to recommend (LTR) as well as with two additional questions to find about the simulator perceived utility by the students. Interestingly, we found that better scores were obtained when students were provided with more guidance about the simulator and with more simulator-oriented exercises. In addition, the best scores were obtained in the classroom experience described in this paper, as opposed to the on-line experiences. The 2019 experience, however, received better scores than the one in the previous year and with figures approaching those of the classroom experience here described (LTR were above 4 in both cases, in a 1–5 scale, while the one in 2018 did not exceed 3.7). Complete results can be found in [21,22].

This study has the obvious limitation of a short range evaluation, built on the basis of a one-day experience, so our formal conclusions do not carry over to long-term simulation-based training. On the other hand, and as a positive feature, we have avoided the onset of any type of contamination between the CG and the EG. Despite this is the case, the two on-line experiences described in the previous paragraph provided us with some insight about student satisfaction on longer experiences, since the courses were approximately 50 h long. While in the on-line cases no CG and EG groups were defined about the use of the simulator—in 2019 the groups were split for either to use the simulator with an integrated intelligent tutoring system or without it—and, consequently, no hypothesis test could be accomplished, our results indicate that the simulator is indeed satisfactory for the students in a long-term course. SUS scores, however, were moderate, but this is probably due to the fact that the simulator deals with complex ideas so the simulator should not hide such a complexity from the students, since once the students become professionals they should deal with interfaces similar to the one we have built.

## 5. Conclusions

In this work we have presented the evaluation of an education-oriented MRI computer simulation paradigm, grounded on a number of functional and non-functional requirements. We have designed an experiment aimed at comparing the simulator performance with that obtained using a traditional educational approach. All in all, we have no evidence that any sort of educational evaluation study in this application domain has been described in the literature, so ours seems to be the first attempt to shed some light on this important topic. Our results show that such an educational tool has given rise to an improvement in the applied learning outcomes, so we conclude that the tool is indeed useful for gaining dexterity in the MRI acquisition process.

## Figures and Tables

**Table 1:** Statistical analysis of the pre-test for the 10-item instrument. The first part of the table (Hits) uses the hits rating while the second one (NE) uses the null-expectation rating. HT: Hypothesis test; Med: Median; SD: Standard deviation; E. size: Effect size.

**Hits**	**HT**	**Exp. Group**		**Ctrl. Group**		**E. Size**
	**(*p*-Val)**	**Med**	**Mean ± S D**	**Med**	**Mean ± SD**	**(Cohen’s d)**
**T**	0.588	0.50	1.13 ± 1.28	0.50	0.90 ± 1.16	0.191
**P**	0.938	0.00	0.53 ± 0.82	0.00	0.57 ± 0.86	−0.040
**NE**	**HT**	**Exp. Group**		**Ctrl. Group**		**E. Size**
	**(** ***p*** **-Val)**	**Med**	**Mean ± SD**	**Med**	**Mean ± SD**	**(Cohen’s d)**
**T**	0.552	0.33	0.74 ± 1.29	0.00	0.51 ± 1.23	0.185
**P**	0.915	0.00	0.13 ± 0.88	0.00	0.22 ± 0.98	−0.096

**Table 2:** Statistical analysis of the post-test and the gain for the 10-item instrument. The first part of the table (Hits) uses the hits rating while the second one (NE) uses the null-expectation rating. PT: Post-test; HT: Hypothesis test; Med: Median; SD: Standard deviation; E. size: Effect size.

**Hits**		**HT**	**Exp. Group**		**Ctrl. Group**		**E. Size**
		**(*p*-Val)**	**Med**	**Mean ± SD**	**Med**	**Mean ± S**D	**(Cohen’s d)**
**PT**	**T**	0.540	1.50	1.93 ± 1.48	2.00	2.17 ± 1.51	−0.156
	**P**	**0.018**	2.00	2.53 ± 1.36	2.00	1.63 ± 1.27	**0.684**
**Gain**	**T**	0.309	1.00	0.80 ± 1.27	1.00	1.27 ± 1.48	-0.338
	**P**	**0.036**	2.00	2.00 ± 1.62	1.00	1.07 ± 1.36	**0.624**
**NE**		**HT**	**Exp. Group**		**Ctrl. Group**		**E. Size**
		**(** ***p*** **-Val)**	**Med**	**Mean ± SD**	**Med**	**Mean ± SD**	**(Cohen’s d)**
**PT**	**T**	0.581	0.83	1.28 ± 1.78	1.17	1.54 ± 1.94	−0.143
	**P**	**0.013**	1.83	1.99 ± 1.67	1.17	0.93 ± 1.55	**0.655**
**Gain**	**T**	0.29	0.67	0.53 ± 1.67	1.00	1.03 ± 2.66	−0.276
	**P**	**0.01**	1.67	1.86 ± 1.79	0.67	0.71 ± 1.95	**0.686**

**Figure A1:**
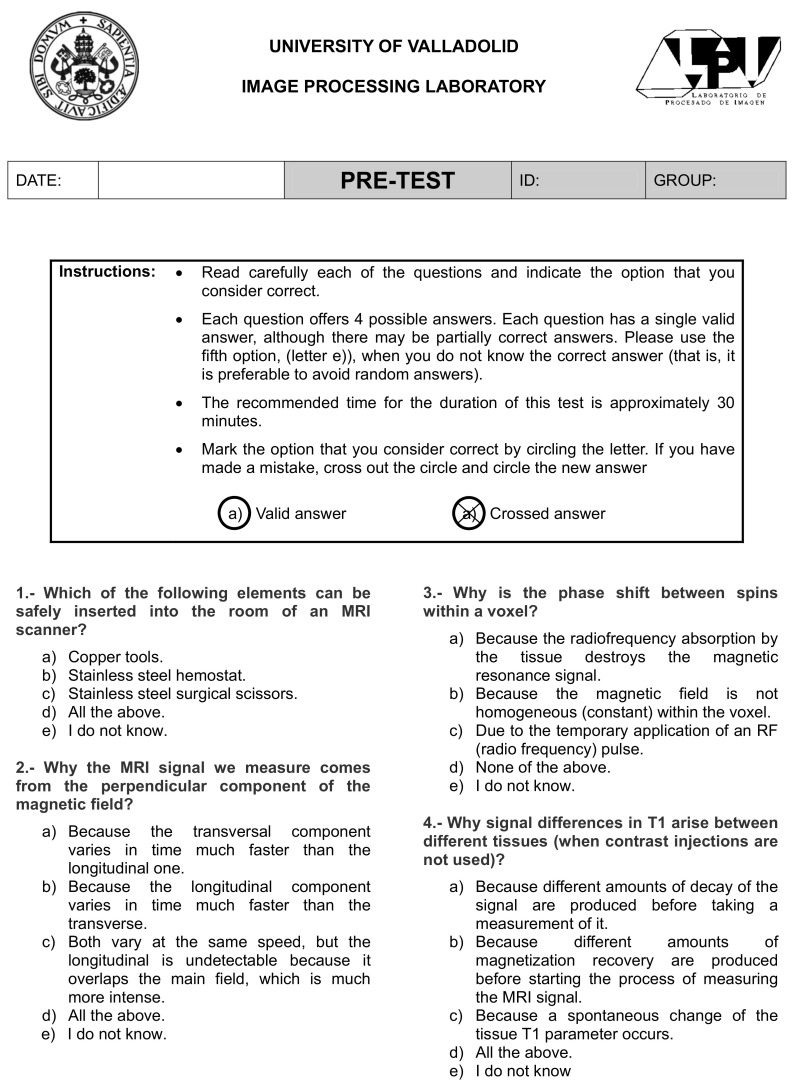
Instrument page 1 of 4.

**Figure A2:**
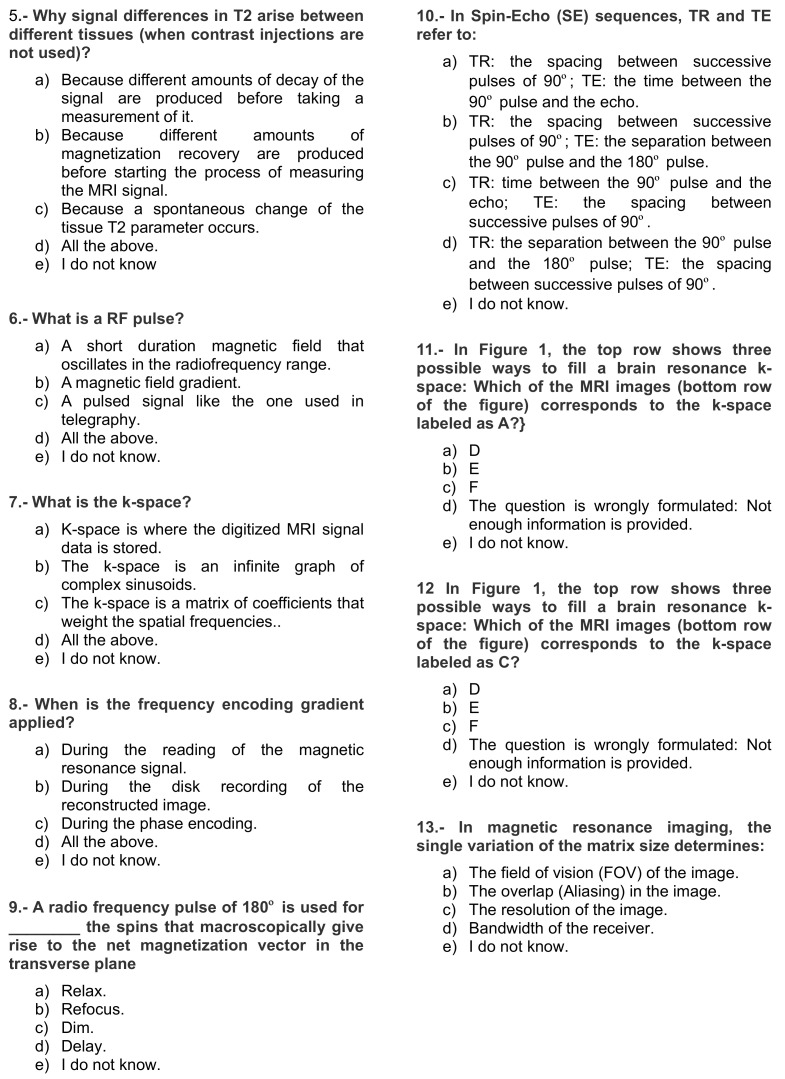
Instrument page 2 of 4.

**Figure A3:**
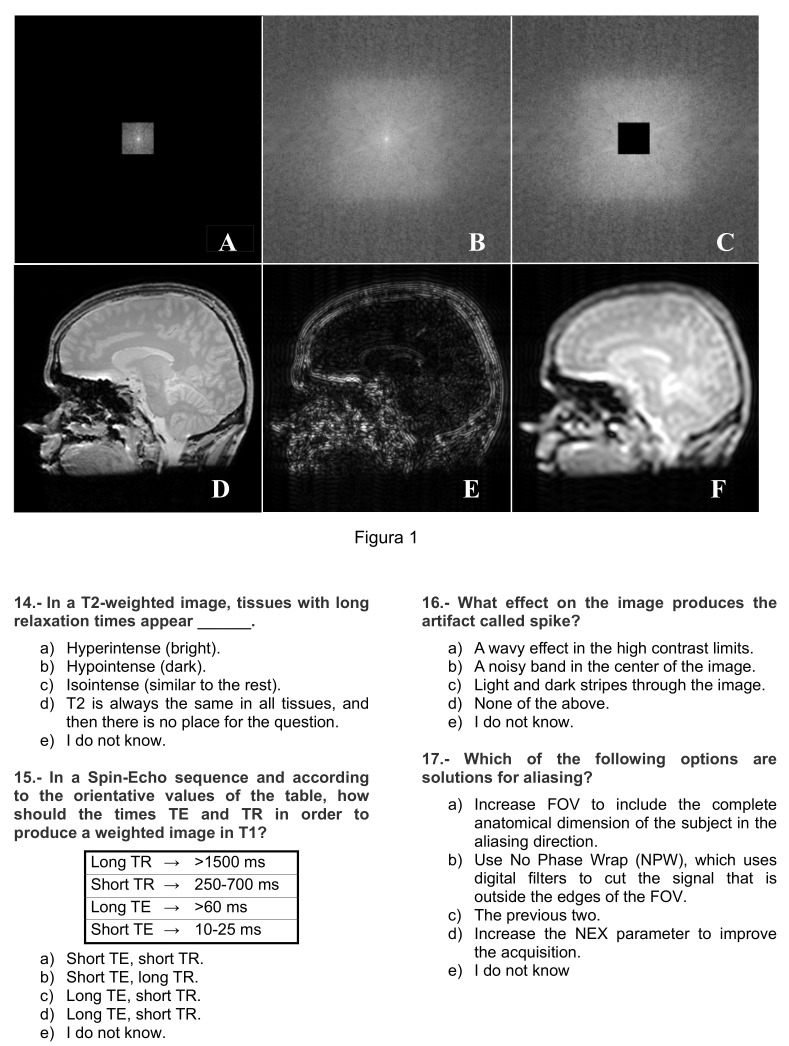
Instrument page 3 of 4.

**Figure A4:**
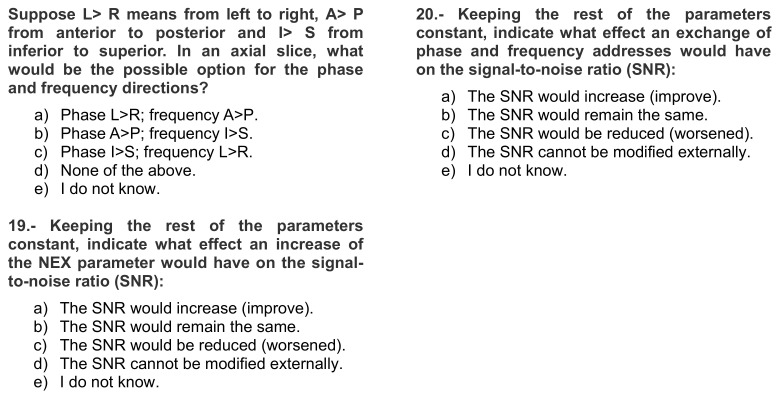
Instrument page 4 of 4.

## Data Availability

The datasets generated during the current study are available at http://www.lpi.tel.uva.es/~carlos/mriSimuExp.xlsx (last access on 30 July 2021). The file contains the anonymized test results. It is divided into 23 columns, the three initial columns are: The identifier of the participant, the test (pre-test or post-tets) and the group to which it belongs (control or experimental.) The other columns contain responses to the questions posed in the instrument, where each point is a hit, each one third negative point is an error, and the none point means the “I do not know” answer.

## Abbreviations

The following abbreviations are used in this manuscript:

$\Delta B_{0}$	field inhomogeinity
API	Application Program Interface
CG	Control Group
EG	Experimental Group
ETL	Echo Train Length
FOV	Field Of View
GE	Gradient-Echo
GUI	Graphical User Interface
MRI	Magnetic Resonance Imaging
MVVM	Model-View-ViewModel
NE	Null Expectation
P part	Practical part
PD	Proton Density
REST	Representational State Transfer
SD	Standard Deviation
SE	Spin-Echo
SOA	Service-Oriented Architecture
SOAP	Simple Access Protocol

T part	Theoretical part
$T_{1}$	Longitudinal Relaxation Time
$T_{2}$	Transverse Relaxation Time
$T_{E}$	Echo Time
$T_{I}$	Inversion Time
$T_{R}$	Repetition Time

## Appendix A. Simulation Engine Implementation Details

In this appendix we will describe additional details on the simulation engine. Figure 3 is used as a reference for our description.

### Appendix A.1. Anatomical Model and Model Re-Slicing

The simulator has a database of models from different parts of the human anatomy. They have been obtained from volunteers after executing mapping sequences with submilimetric resolutions and applying relaxometry procedures [34,35]. For each model we have the $T_{1}$, $T_{2}$ and proton density maps, $T_{1}\left(x\right)$, $T_{2}\left(x\right)$ and ρ(x) respectively, where x is a discrete variable defined within some discrete domain, say, $X_{d}$, which, in turn, is a subset of some continuous domain X, i.e., $x\in X_{d}\subset X\subset R^{3}$. Hence, each model consists of a 3D volume where for each x we have the three-component vector $V\left(x\right)=\left[T_{1}\left(x\right),T_{2}\left(x\right),PD\left(x\right)\right]$.

The spatial planning consists in defining planes in any desired direction; then the values V(y), with y∈X belonging to one of these planes, are obtained by interpolating the values V(x) with $x\in N_{y}$, and $N_{y}\in X_{d}$ a neighborhood of y at the voxel size selected by the user. Each map is interpolated only from points from the same map.

Slice thickness is simulated by defining a slab of the thickness set by the user. This slab will contain, generally speaking, several slices of the original or the interpolated volume. The slices will be Gaussian filtered in the orthogonal direction and summed to the give rise to the slab values.

### Appendix A.2. Image Contrast

Assuming we have the map values at position x⊂X, which may be either original or interpolated as indicated above, well-known expressions of commonly-used sequences are evaluated on the values V(x) to simulate image contrast. These three examples [25]:

$$\begin{array}{cc} I_{SE-SR}\left(x\right) & =\rho \left(x\right)\left(1-2e^{-\frac{T_{R}-T_{E}/2}{T_{1}\left(x\right)}}+e^{-\frac{T_{R}}{T_{1}\left(x\right)}}\right)e^{-\frac{T_{E}}{T_{2}\left(x\right)}}\\ I_{SE-IR}\left(x\right) & =\rho \left(x\right)\left(1-2e^{-\frac{T_{I}}{T_{1}\left(x\right)}}+2e^{-\frac{T_{R}-T_{E}/2}{T_{1}\left(x\right)}}-e^{-\frac{T_{R}}{T_{1}\left(x\right)}}\right)e^{-\frac{T_{E}}{T_{2}\left(x\right)}}\\ I_{GE}\left(x\right) & =\rho \left(x\right)\frac{\left(1-e^{-\frac{T_{R}}{T_{1}\left(x\right)}}\right)}{1-\cos\left(\alpha \right)e^{-\frac{T_{R}}{T_{1}\left(x\right)}}}\sin\left(\alpha \right)e^{-\frac{T_{E}}{T_{2}^{*}\left(x\right)}} \end{array}$$

respectively approximate SE with saturation recovery, SE with inversion recovery and fast GE with crusher gradients. The parameters involved, apart from the three maps we have described above, are $T_{R}$ (repetition time), $T_{E}$ (echo time), $T_{I}$ (inversion time) and α (flip angle). These parameters will be set by the user at will, within allowable ranges. Parameter $T_{2}^{*}$ is version of $T_{2}$ modified by field inhomogeneity, where the relation between both is

$$\frac{1}{T_{2}^{*}\left(x\right)}=\frac{1}{T_{2}\left(x\right)}+\gamma \Delta B_{0}\left(x\right)$$

with γ the gyromagnetic constant [25] and $\Delta B_{0}\left(x\right)$ an assumed field heterogeneity model; in our current implementation it is just a constant field aimed at illustrating the difference between spin and gradient echo sequences in terms of attenuation. Fast SE sequences, in which multiple echoes are obtained per TR interval, are simulated as follows: we define

$$T_{E}\left(n\right)=T_{E_{eff}}-\left(\left\lfloor \frac{ETL-1}{2}\right\rfloor -n\right),n=0,1,\ldots ,ETL-1$$

with ETL the echo train length, i.e., the number of echoes per TR, $T_{E_{eff}}$ the $T_{E}$ in which the k-space centerline is read and $T_{E}\left(n\right)$ the echo time that corresponds to the *n*-th segment used to fill up k-space. Then, as many images per slice as ETL are calculated and the fraction of k-space lines that correspond to the *n*-segment are used to fill the k-space of the fast spin echo sequence. The image is then Fourier inverse transformed.

Images are simulated in image space using these expressions and are then transformed to k-space domain for artifact inclusion. If partial Fourier is selected, only the percentage of k-space selected by the user is populated, while the remaining part is filled by conjugate symmetry.

### Appendix A.3. Spatial Planning and Fold over Effects

The simulator allows the user to spatially plan the images to simulate as well as to select a field of view (FOV). In the case that the FOV is not selected properly, i.e., excited areas of the volume are left outside of the FOV, a well known fold over effect takes place. This is easily simulated by carrying our a fast Fourier transform of the selected slice, and then carrying out a periodic extension of this transform with a separation between two replicas equal to the length of the FOV in the phase coding direction.

The simulator offers different ways of avoiding this effect. One of them is to use saturation bands that eliminate signal from out-of-FOV areas. To this end, the user has to set the option of saturation bands and to place these bands properly. A second one is the no phase wrap which enlarges the FOV as for acquisition although for visualization the original FOV is shown.

### Appendix A.4. Motion and Noise

Motion is simulated by means of assuming a motion model that takes place through the acquisition. k-space lines acquired in different motion states are put together and then the acquisition is transformed back with incoherent lines. Specifically, the motion model is a rotation with an angle span equal to ±5 degrees and k space lines are segmented in five angulations, with values [−5,−2.5,0,2.5,5].

As for noise, termal, radiofrequency and spike noise models are available. As for termal noise, white Guassian noise is added in k-space. The variance of the noise has been predefined for a canonical acquisition. Then this variance is modified taking into account the resolution of the image (i.e., the pixel size) as well as the number of phase encodings and the bandwith, using well-known dependencies [26]. NEX is simulated by repeating the acquisition and direct averaging in k-space.

Spikes and RF noise are simulated by enlarging the image spectrum value(s) at one point in k-space for the former or at one line for the latter. The point is selected within a line $\frac{1}{8}$ away from the center of k-space relative to the Fourier transform width of the original image in the phase encoding dimension and $\frac{1}{9}$ in the frequency encoding direction. RF contamination selects the whole line $\frac{1}{6}$ away from the center in the phase direction. The multiplication factor has a module about 20.
